# CNP-pGC-cGMP-PDE3-cAMP Signal Pathway Upregulated in Gastric Smooth Muscle of Diabetic Rats

**DOI:** 10.1155/2015/305258

**Published:** 2015-03-25

**Authors:** Ying-Lan Cai, Mo-Han Zhang, Xu Huang, Jing-Zhi Jiang, Li-Hua Piao, Zheng Jin, Wen-Xie Xu

**Affiliations:** ^1^Department of Physiology, Yanbian University School of Medicine, 977 Gongyuan Road, Yanji, Jilin 133002, China; ^2^Department of Physiology, Shanghai Jiaotong University School of Medicine, 800 Dongchuan Road, 328 Wenxuan Medical Building, Shanghai 200240, China

## Abstract

Our previous studies have shown that CNP-NPR-B/pGC-cGMP is upregulated in the diabetic rats. The present study was designed to determine whether the upregulation of CNP-NPR-B/pGC-cGMP signal pathway affects cGMP-PDE3-cAMP signal pathway in diabetic gastric smooth muscle. The gastric smooth muscle motility was observed by using isometric measurement. PDEs expressions in diabetic gastric smooth muscle tissue were observed by using immunohistochemistry, Western blotting, and RT-PCR methods. The results demonstrated that the inhibitory effect of CNP on the spontaneous contraction of gastric antral circular smooth muscle was potentiated in STZ-induced diabetic rat. CNP-induced increase of cGMP and cAMP was much higher in diabetic gastric smooth muscle tissue than in controls. The expression of PDE3 is downregulated while the levels of gene expression of PDE1, PDE2, PDE4, and PDE5 were not altered in the diabetic gastric smooth muscle tissue. The results suggest that the sensitivity of gastric smooth muscle to CNP is potentiated via activation of CNP-pGC-cGMP-PDE3-cAMP signal pathway in STZ-induced diabetic rats, which may be associated with diabetes-induced gastric motility disorder.

## 1. Introduction

Gastrointestinal motility disorders are common complications of diabetes, which can happen in all regions of the gastrointestinal tract. It has been reported that 75% of patients with diabetes are often accompanied by gastrointestinal symptoms, such as abdominal distention, epigastric discomfort, and constipation [1]. Diabetic gastrointestinal motility disorders and gastric emptying delay significantly affect the pharmacokinetics of hypoglycemic and other drugs, resulting in absorption delay of these drugs and poor glycemic control. Meanwhile, the delay of absorption probably leads to hypoglycemia, which also affects the curative effect of hypoglycemic drugs. However, the mechanism of diabetes-induced gastric motility disorders is still unclear [1, 2].

C-type natriuretic peptide (CNP) is an important member of natriuretic peptides (NPs) family, which was firstly discovered in the gastrointestinal tract by Komatsu et al. [3] in 1991. Then Gower Jr. et al. [4] found three kinds of natriuretic peptide receptors (NPRs) in the mucosa and smooth muscle tissues of gastric antrum in 2001, which were named NPR-A, NPR-B, and NPR-C. The NPs-NPR-A, B/pGC-cGMP signal pathway was found to be involved in the inhibitory effect of NPs on spontaneous contraction in gastric smooth muscle of guinea pig, rat, and human [5, 6]. Elevation of intracellular cAMP and cGMP has been associated with smooth muscle relaxation in several regions of the gastrointestinal tract, including the lower oesophageal sphincter, ileum, proximal colon, taenia coli, and internal anal sphincter [7–13]. The intracellular levels of cAMP and cGMP reflect a balance between their synthesis and catabolism, the latter being regulated by the enzymes of phosphodiesterase (PDE) family. At least five families, designated PDEs 1–5, have been classified according to their substrate preference, cofactor requirements, and sensitivity to endogenous inhibitors and activators [14–16]. In the heart, PDE1, PDE2, PDE3, PDE4, PDE5, PDE8, and PDE9 have been described. PDE1, PDE2, and PDE3 can hydrolyze both cAMP and cGMP, whereas PDE4 and PDE8 are selective for cAMP, and PDE5 and PDE9 are selective for cGMP. However, cGMP can inhibit cAMP hydrolysis by PDE3 and possibly PDE1, whereas cGMP can activate PDE2 [17].

We have found that both NPR-B expression in smooth muscle of gastric antrum and the activity of membrane-bound guanylate cyclase (pGC) were significantly increased in diabetic rats, which indicate the upregulation of CNP-NPR-B/pGC-cGMP signal pathway in diabetic gastric antrum [18, 19]. And we also found that cGMP produced through CNP-NPR signal pathway induced the generation of cAMP via cGMP-PDE3-cAMP signal pathway followed by the activation of PKA resulting in the inhibition of L-type calcium current, which inhibited the spontaneous contraction of gastric smooth muscle together with cGMP [20].

According to our above studies, we are wondering whether the upregulation of CNP-NPR-B/pGC-cGMP signal pathway affects cGMP-PDE3-cAMP signal pathway in diabetic animals. In the present study, we used the diabetic rat model induced by streptozotocin (STZ) to explore the activity of cGMP-PDE3-cAMP signal pathway in the gastric antrum.

## 2. Materials and Methods

### 2.1. Animals and STZ-Induced Diabetic Rat Model

Wistar rats (either sex, weighed 220~250 g) used in the present study were provided by Experimental Animal Center of Yanbian University. Animals were fasted overnight with* ad libitum* access to water before intraperitoneal administration of STZ (Sigma-Aldrich, St. Louis, MO, USA), which was freshly prepared in citrate buffer at a dose of 65 mg/Kg body weight to produce diabetic model. Control group was intraperitoneally administered the same volume of citrate buffer. One week after administration, blood was withdrawn from the rat tail vein to measure glucose concentration. Diabetes was defined when the blood glucose level was above 350 mg/dL. The control and diabetic animals were raised separately with free access to food and water for four weeks.

### 2.2. Preparation of Muscle Strips and Isometric Tension Measurement

Rats were anaesthetized and the stomachs were removed quickly and placed in aerated (95% O_2_ and 5% CO_2_) Krebs solution which contains (mM) NaCl 118, KCl 4.75, CaCl_2_ 2.54, KH_2_PO_4_ 1.19, MgSO_4_ 1.19, NaHCO_3_ 25, and glucose 10. The stomach was cut along the lesser curvature and the mucosa was removed carefully. The muscle strips (2 × 12 mm) were cut along the circular axis of the antrum and then mounted in a vertical organ bath containing oxygenated Krebs solution maintained at 37°C. One end of the strip was fixed onto a platinum hook, and the other end was fixed onto an isometric force transducer connected to the RM6240C biological signal processing system (Chengdu Equipment Factory, Chengdu, China) to record the mechanical activity. A tension of 1 g was applied to the tissues and they were equilibrated for 40 min before the experiments when rhythmic spontaneous contractions were recorded. All experimental protocols performed were approved by the local Animal Care Committee and conformed to the Guide for the Care and Use of Laboratory Animals published by the Science and Technology Commission of China (STCC Publication number 2, revised 1988).

After recording the baseline contraction of control or diabetic rats, 0.1 *μ*M CNP (Sigma-Aldrich, St. Louis, MO, USA) was added to the organ bath. The amplitude and frequency of contraction before and after administration of CNP were recorded for 5 min to observe the sensitivity of gastric smooth muscle to CNP in control and diabetic rats.

The bath solution after administration was collected and frozen in liquid nitrogen and then kept at −80°C for later analysis of the content of cAMP and cGMP.

### 2.3. cGMP and cAMP Content

The bath solution of 300 *μ*L was treated with 300 *μ*L 6% triethylamine (TCA) and then incubated for 15 min at room temperature. The samples were washed three times with water-saturated ethyl ether and lyophilized with lyophilizer (Savant, Farmingdale, NY). The intracellular contents of cGMP and cAMP in the samples were determined using cGMP or cAMP Direct Immunoassay Kit (BioVision Research Products, USA) after being reconstituted in the assay buffer. The standards and samples were acetylated firstly according to the manufacturer's instructions to increase the sensitivity of the assay.

### 2.4. Immunohistochemistry

The stomachs of control and diabetic rats were obtained as described above. After being washed with physiological saline to remove the gastric content, the antrum was fixed with 4% paraformaldehyde for 24 h at 4°C and dehydrated in ethanol and then embedded with paraffin. The sections were cut, mounted on glass slides, and dried overnight. After being deparaffinized with xylene and rehydrated in ethanol, the sections were washed in PBS for 5 min. Endogenous peroxidase activity was blocked with incubation in 3% hydrogen peroxide at room temperature for 30 min. The sections were then incubated at 100°C in 10 mmoL/L citrate buffer (pH 6.0) for 10 min to retrieve antigens, cooled for 20 min, and then washed in PBS. After being incubated in PBS containing 10% normal goat serum at 37°C for 45 min, the sections were incubated with rabbit anti-PDE2 antibody (1 : 500, Abcam, Cambridge, MA, USA) or rabbit anti-PDE3 antibody (1 : 500, Abcam, Cambridge, MA, USA) at 4°C overnight. After washing, the sections were incubated with biotinylated goat anti-rabbit IgG at 37°C for 30 min, followed by incubation with streptavidin-horseradish peroxidase at 37°C for 30 min. 3,3′-Diaminobenzidine was used as a chromogen and hematoxylin was used for counterstaining. The sections for which the primary antibodies were omitted in the same procedure were used as controls.

### 2.5. Western Blotting Analysis

Rat antral tissues obtained from the protocol described above were homogenized in RIPA lysis buffer (Beyotime, Jiangsu, China) supplemented with 1 mM PMSF (Beyotime, Jiangsu, China) on ice. The homogenate was then centrifuged at 12,000 ×g for 15 min at 4°C. After determining the protein concentration, the supernatant was mixed with loading buffer and heat denatured at 100°C for 10 min. An equal amount of protein (30 *μ*g) of each sample was separated by 10% SDS-PAGE gel electrophoresis and then electrotransferred onto a nitrocellulose membrane (Amersham Pharmacia Biotech, Piscataway, NJ, USA). The membranes were blocked with 5% non-fat dry milk in Tris-buffered saline (TBS, pH 7.6) containing 0.5% Tween-20 for 2 h at room temperature and then incubated with a primary rabbit anti-PDE2 antibody (1 : 500, Abcam, Cambridge, MA, USA), rabbit anti-PDE3 antibody (1 : 500, Abcam, Cambridge, MA, USA), or mouse anti-*β*-actin antibody (1 : 10000, Abcam, Cambridge, MA, USA) at 4°C overnight. After being washed with TBST three times (15 min each), the membranes were incubated with HRP-labeled goat anti-rabbit IgG or HRP-labeled goat anti-mouse IgG at room temperature for 2 h. 3,3′-Diaminobenzidine was used as a chromogen. The image from each Western blot was quantitatively analyzed using Quantity One software (Bio-Rad) and normalized by that of *β*-actin.

### 2.6. Reverse Transcriptase Polymerase Chain Reaction (RT-PCR) Analysis of PDE1, PDE2, PDE3, PDE4, and PDE5 Gene Expression

Tissues of the rat stomach were obtained as described above. Total RNA was extracted from the whole stomach according to the manufacturer's instructions for the TRIzol Reagent (TaKara, Japan). RNA concentration was determined by absorbance reading at 260/280 nm which was between 1.8 and 2.0. Primer sequences for rat PDE1, PDE2, PDE3, PDE4, PDE5, and GAPDH were as follows: PDE1A (sense) 5′-AGATGACTGGAGGGATCTTCGG-3′, PDE1A (antisense) 5′-AGCTTCCACGTTTTGGCTGG-3′; PDE2A (sense) 5′-GATCAAAAGGATGAGAAGGG-3′, PDE2A (antisense) 5′-TTGCACATCGTCAGAGGTTAGG-3′; PDE3A (sense) 5′-TGAGACCAACAACAACAGTGA-3′, PDE3A (antisense) 5′-GAGTATAGGTGCCACAAGCC-3′; PDE4A (sense) 5′-GCGGGACCTACTGAAGAAATTCC-3′, PDE4A (antisense) 5′-CAGGGTGGTCCACATCGTGG-3′; PDE5A (sense) 5′-AACACGCACTGCATCAGAAG-3′, PDE5A (antisense) 5′-CGCTGTTTCCAGATCAGACA-3′; GAPDH (sense) 5′-GATTTGGCCGTATCGGAC-3′, GAPDH (antisense) 5′-GAAGACGCCAGTAGACTC-3′. Reverse transcription was performed according to the manufacturer's instructions. The following conditions were used for PCR amplification: 95°C for 5 min, followed by 30 cycles at 95°C for 30 sec; 58~60°C for 1 min; 72°C for 2 min, followed by 72°C for 10 min. The PCR products were separated on a 1% agarose gel. Detectable fluorescent bands were visualized by an ultraviolet transilluminator (Bio-Rad) and quantified using Quantity One image software. The mRNA expression level for PDE1, PDE2, PDE3, PDE4, and PDE5 was normalized by that of GAPDH.

### 2.7. Data Analysis

The average amplitude and frequency of the contraction recorded before and after administration of drugs were considered as control and effect size, respectively. The percentage of the change was expressed as (effect size − control)/control × 100%. The level of the baseline was considered as the basal tension (g), which was 0 g before treatment. The tension after treatment was expressed as the change of baseline (g)/weight of the muscle strip (g). The duration from the amplitude of contraction suppressed to minimum by CNP to contraction recovery was considered as the time of complete inhibition of CNP. Data was analyzed using Origin 6.0 software and expressed as means ± SEM. Data recordings were evaluated using a Student's *t*-test. *P* values less than 0.05 were considered statistically significant.

## 3. Results

### 3.1. Changes in Blood Glucose Concentration

Four weeks after injection of STZ, all the animals treated with STZ exhibited hyperglycemia. The mean blood glucose concentration of animals defined as diabetes was 455.2 ± 32.8 mg/dL, which was significantly higher than controls (106.4 ± 16.9 mg/dL; *P* < 0.05).

### 3.2. Effect of CNP on the Spontaneous Contraction of Gastric Smooth Muscle

**Figure 1:**
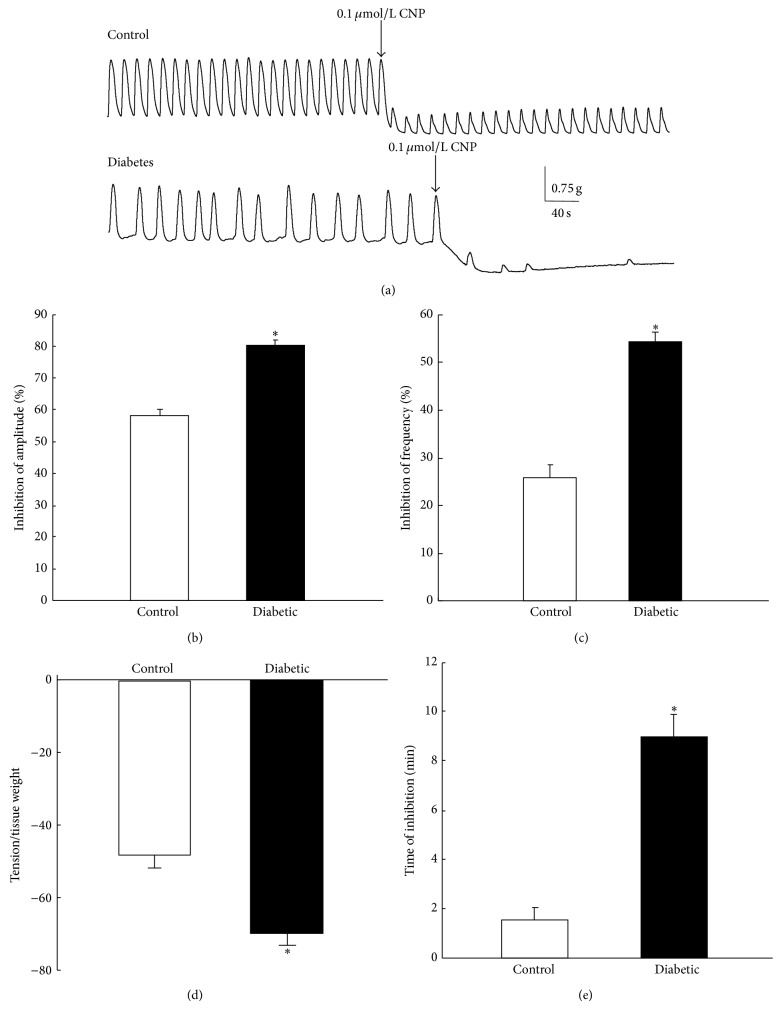
Effect of CNP on the spontaneous contraction of gastric antral smooth muscle in control and diabetic rats. (a) CNP significantly inhibited the spontaneous contraction of gastric antral smooth muscle in both control and diabetic rats. (b) CNP caused significantly more inhibition on the amplitude of the contraction in diabetic rats than in controls (*n* = 8, *P* < 0.01). (c) CNP caused significantly more inhibition on the frequency of the contraction in diabetic rats than in controls (*n* = 8, *P* < 0.01). (d) CNP decreased the basal tension, which was more significant in diabetic rats than in controls (*n* = 8, *P* < 0.01). (e) The duration of CNP-induced inhibition of gastric smooth muscle contraction was significantly longer in diabetic rats than in controls (*n* = 8, *P* < 0.01).

After equilibration, spontaneous contractions of gastric antral circular smooth muscle from both control and diabetic rats were recorded. CNP (0.1 *μ*mol/L) significantly inhibited the spontaneous contractions in both control and diabetic rats; however, the inhibitory response to CNP was much stronger in diabetic group (Figure 1(a)). CNP (0.1 *μ*mol/L) inhibited the amplitudes of the contraction by 57.92  ±  4.66% in control rats and 80.15  ±  3.10% in diabetic rats (Figure 1(b), *n* = 8, *P* < 0.01). CNP (0.1 *μ*mol/L) inhibited the frequencies of the contraction by 25.85 ± 6.57% in controls and 54.37 ± 5.35% in diabetic rats (Figure 1(c), *n* = 8, *P* < 0.01). Meanwhile, the tensions were significantly decreased to 47.88 ± 3.62 and 70.05 ± 3.21 after the administration of CNP in normal and diabetic groups, respectively (Figure 1(d), *n* = 8, *P* < 0.01). The durations of CNP-induced inhibition were 1.52 ± 0.50 min in control and 8.98 ± 0.88 min in diabetic group (Figure 1(e), *n* = 8, *P* < 0.01). The results suggest that the inhibitory effect of CNP on spontaneous contraction is potentiated in diabetic rats.

### 3.3. Effect of CNP on cGMP and cAMP Generations in Gastric Smooth Muscle

**Figure 2:**
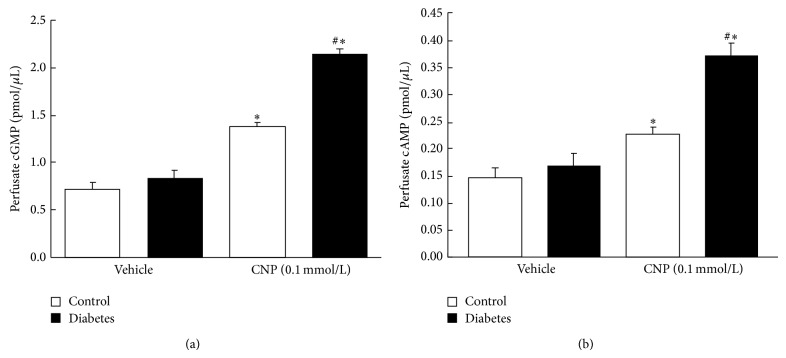
Effect of CNP on intracellular cGMP and cAMP levels in gastric smooth muscle of diabetic and control rats. (a) CNP increased intracellular cGMP level in gastric smooth muscle, which was more significant in the diabetic rats than in controls (*n* = 8, ^#^ *P* < 0.01 versus vehicle, ^*^ *P* < 0.01 versus control). (b) CNP increased intracellular cAMP level in gastric smooth muscle, which was more significant in the diabetic rats than in controls (*n* = 8, ^#^ *P* < 0.01 versus vehicle, ^*^ *P* < 0.01 versus control).

CNP binds to the NPR-A or NPR-B in smooth muscle cell membrane and causes the production of cGMP by activating pGC. Since the inhibitory effect of CNP on spontaneous contraction was potentiated in diabetic rats, in succession, the effects of CNP on cGMP and cAMP generations were observed. Our experiments demonstrated that CNP significantly increased intracellular cGMP and cAMP concentrations in the gastric smooth muscles of both control and diabetic rats. cGMP and cAMP productions in control rats were increased from 0.71 ± 0.09 pmol/*μ*L and 0.15 ± 0.05 pmol/*μ*L before to 1.37 ± 0.12 pmol/*μ*L and 0.23 ± 0.03 pmol/*μ*L after the administration of CNP, respectively, and the increase percentage was 93.78% and 56.16%, respectively (Figures 2(a) and 2(b), *n* = 8, *P* < 0.01), while, in the diabetic group, the cGMP and cAMP productions were increased from 0.83 ± 0.22 pmol/*μ*L and 0.17 ± 0.05 pmol/*μ*L before to 2.15 ± 0.16 pmol/*μ*L and 0.37 ± 0.07 pmol/*μ*L after the administration of CNP, respectively, and the increase percentage was 159% and 118%, respectively (Figures 2(a) and 2(b), *n* = 8, *P* < 0.01). The CNP-induced productions of cGMP and cAMP in gastric smooth muscle were significantly potentiated in diabetic rats.

### 3.4. Expressions of PDEs in the Gastric Smooth Muscle

**Figure 3:**
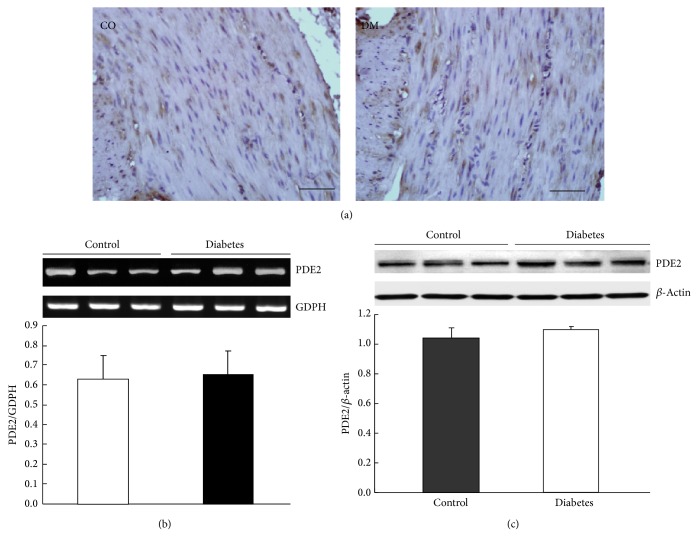
Expression of PDE2 in the gastric smooth muscle. (a) PDE2 immunoreactivity in gastric smooth muscle was not different between the diabetic rats and controls (bars = 20 *μ*m). (b) The mRNA level of PDE2 in the gastric smooth muscle was not different between diabetic rats and controls (*n* = 9, *P* > 0.05). (c) The protein level of PDE2 detected by Western blotting analysis in the gastric smooth muscle was not significantly different between diabetic rats and controls (*n* = 9, *P* > 0.05).

**Figure 4:**
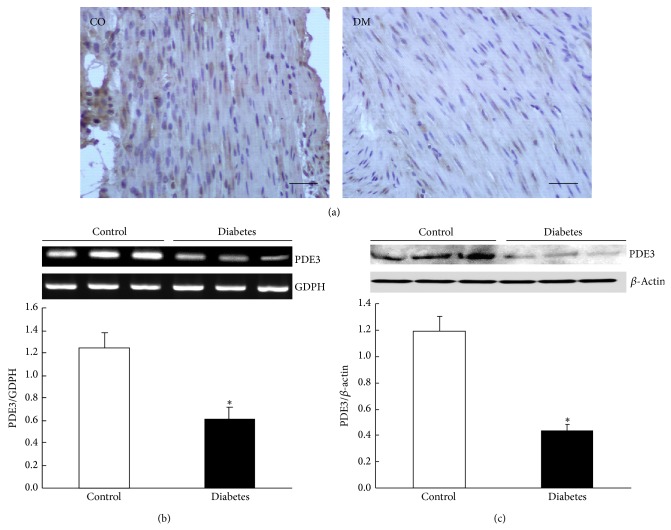
Expression of PDE3 in the gastric smooth muscle. (a) PDE3 immunoreactivity in gastric smooth muscle was lower in the diabetic rats than in controls (bars = 20 *μ*m). (b) The mRNA level of PDE3 in the gastric smooth muscle was downregulated in the diabetic rats (*n* = 9, *P* < 0.05). (c) The protein level of PDE3 detected by Western blotting analysis in the gastric smooth muscle was significantly lower in the diabetic rats than in controls (*n* = 9, *P* < 0.05).

**Figure 5:**
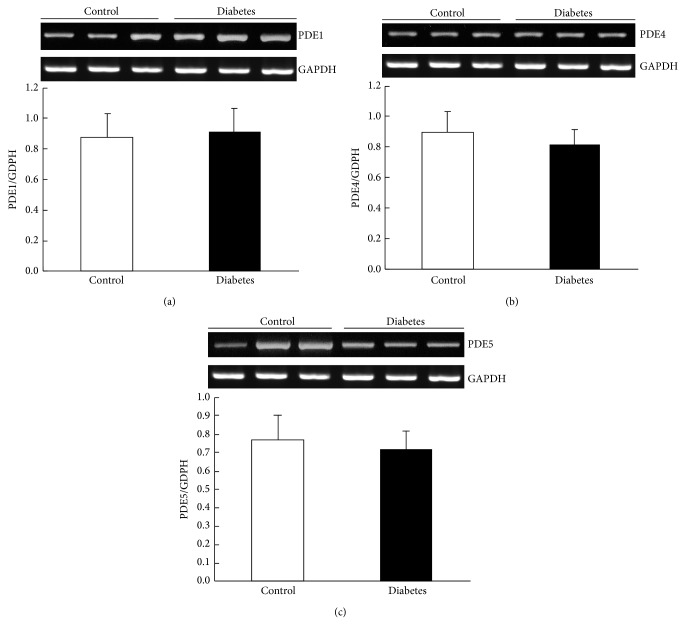
Expression of other PDEs in the gastric smooth muscle. (a) The mRNA level of PDE1 in the gastric smooth muscle was not different between the diabetic rats and controls (*n* = 9, *P* > 0.05). (b) The mRNA level of PDE4 in the gastric smooth muscle was not different between diabetic rats and controls (*n* = 9, *P* > 0.05). (c) The mRNA level of PDE5 in the gastric smooth muscle was not different between diabetic rats and controls (*n* = 9, *P* > 0.05).

The cellular levels of the cyclic nucleotides reflect a balance between their synthesis and catabolism, and the latter was regulated by the enzymes of phosphodiesterase (PDE) family. Our previous study has shown that the generation of cAMP induced by CNP is via CNP-cGMP-PDE3-cAMP signal pathway in control rats [9]. Since the CNP-induced productions of cGMP and cAMP in gastric smooth muscle were significantly potentiated in diabetic rats, we were wondering whether the expression of PDE was changed in the diabetic rats. Firstly, we observed the expressions of PDE2 and PDE3 in the gastric smooth muscle of both control and diabetic rats by using immunohistochemistry technique, RT-PCR, and Western blotting methods. We found that the expression of PDE2 was not significantly different between the two groups. The PDE2 immunoreactive staining of gastric smooth muscle tissue was not different between control and diabetic rats (Figure 3(a)). The relative PDE2 mRNA levels (PDE2/GAPDH) were 0.65 ± 0.12 and 0.63 ± 0.46 in the control and diabetic rats, respectively (Figure 3(b), *n* = 9, *P* > 0.05). At the protein level, the ratios of PDE2/*β*-actin were 1.04 ± 0.07 in control and 1.10 ± 0.02 in diabetic rats (Figure 3(c), *n* = 9, *P* > 0.05). However, PDE3 expression was lower in the diabetic group than in the control group (Figure 4(a)). At the gene level, the ratios of PDE3/GAPDH were 1.25 ± 0.13 and 0.61 ± 0.11 in control and diabetic rats, respectively (Figure 4(b), *n* = 9, *P* < 0.05), and at the protein level, the ratios of PDE3/*β*-actin were 1.19  ±  0.11 and 0.43 ± 0.05 in control and diabetic rats, respectively (Figure 4(c), *n* = 9, *P* < 0.05). To further determine whether the expressions of other PDEs were changed in diabetic rat stomach, we also observed the expressions of PDE1, PDE4, and PDE5 in the gastric smooth muscle of both normal and diabetic rats. The results demonstrated that the mRNA levels of PDE1, PDE4, and PDE5 were not changed in diabetic rat stomach. The ratios of PDE2/GAPDH were 0.91   ± 0.47, 0.81 ± 0.41, and 0.72 ± 0.07 in control rats, respectively, and 0.88 ± 0.45, 0.90 ± 0.52, and 0.77 ± 0.09 in diabetic rats, respectively (Figure 5, *n* = 9, *P* > 0.05). These results suggest that only the expression of PDE3 was downregulated in gastric smooth muscle tissues of diabetic rats.

## 4. Discussion

NPs as important regulatory peptides in the gastrointestinal tract have been reported to regulate the spontaneous contraction of gastric smooth muscle via NPs-NPR-B/pGC-cGMP signal pathway in the guinea pig, rat, and human [5, 6]. Our previous studies have demonstrated that CNP-NPR-B/pGC-cGMP signal pathway is abnormal in diabetic rats, which is associated with the development of gastric motility disorder [18, 19].

Intracellular cAMP and cGMP are maintained to a proper level through the regulation of their generation and hydrolysis processes. Generation of cAMP and cGMP is catalyzed by the activation of adenylate cyclase and guanylate cyclase, while phosphodiesterase (PDE) is responsible for the hydrolysis of cAMP and cGMP [21, 22]. Via hydrolysis, PDE family can regulate cAMP and cGMP levels, protein phosphorylation, and thus intervene in the signal transduction process, while intracellular cAMP and cGMP can also modulate the activity of PDE. For example, cGMP can increase intracellular cAMP through the inhibition of PDE3 to enhance the cardiac muscle contractility [23, 24]. And we also found that cAMP generation induced by CNP through cGMP-PDE3-cAMP signal pathway activated PKA signal pathway and subsequent inhibition of L-type calcium current to inhibit the gastric smooth muscle motility together with cGMP, while cGMP-PDE2-cAMP signal pathway was not involved in the process [20]. Nevertheless, whether cGMP-PDE-cAMP signal pathway is involved in the mechanism of diabetes-induced gastric motility disorder has not been studied so far.

In the present study, in order to determine the responsiveness of gastric smooth muscle to CNP, we firstly observed the effect of CNP on the spontaneous contraction of gastric antral circular smooth muscle in normal and diabetic rats. The results demonstrated that CNP-induced inhibitory effect was significantly potentiated in diabetic rats (Figure 1). Meanwhile, productions of cGMP and cAMP in gastric smooth muscle induced by CNP were more pronounced in diabetic rats than in controls. These results suggest that the gastric smooth muscle in diabetic rats is more sensitive to CNP than in control rats. In the previous studies, we found that NPR-A, NPR-B, and NPR-C expressions were upregulated in gastric smooth muscle in diabetic mice and rats [18, 19, 25]. Therefore, we suppose that diabetes-induced upregulation of NPRs in gastric smooth muscle potentiates CNP/NPR-A, B/pGC/cGMP-PDE3-cAMP signal pathway.

To further confirm our speculation, we subsequently observed PDEs expressions in gastric smooth muscle of control and diabetic rats by using immunohistochemistry and Western blotting methods. Our results showed that only PDE3 expression in gastric smooth muscle was downregulated in diabetic rats (Figure 4); however, the other PDEs expressions, for example, PDE1, PDE2, PDE4, and PDE5, were not significantly changed in diabetic rats. We have also found that CNP induced relaxation of gastric smooth muscle via CNP/NPR-A,B/pGC/cGMP-PDE3-cAMP signal pathway in normal rats [20]. In heart cGMP can inhibit cAMP hydrolysis by PDE3 and possibly PDE1, whereas cGMP can activate PDE2 [17]. Our results suggest that in gastrointestinal smooth muscle PDE3 may also regulate intracellular cAMP level.

In conclusion, CNP-induced simultaneous increase of cGMP and cAMP productions results in the inhibition of gastric smooth muscle spontaneous contraction in control and STZ-induced diabetic rats. However, diabetes induces upregulation of NPRs/pGC/cGMP signal pathway and the subsequent increase of cGMP is responsible for the downregulation of PDE3 expression, which finally inhibits cAMP hydrolysis and increases intracellular cAMP in gastrointestinal smooth muscle. Our results suggest that CNP/NPR-A, B/pGC/cGMP/PDE3/cAMP signal pathway may be associated with the diabetes-induced gastric motility disorder.
